# Videosensor for the Detection of Unsafe Driving Behavior in the Proximity of Black Spots

**DOI:** 10.3390/s141119926

**Published:** 2014-10-24

**Authors:** Andres Fuentes, Ricardo Fuentes, Enrique Cabello, Cristina Conde, Isaac Martin

**Affiliations:** 1 Universidad de Colima (UCol), Mexico, Ave. Universidad #333, Colonia Las Víboras, Colima 28050, Mexico; E-Mail: fuentesr@ucol.mx; 2 Universidad Rey Juan Carlos (URJC), Escuela Tecnica Superior de Ingenieria Informatica, Tulipán s/n, Móstoles, Madrid 28933, Spain; E-Mails: enrique.cabello@urjc.es(E.C.); cristina.conde@urjc.es(C.C.); isaac.martin@urjc.es(I.M.)

**Keywords:** image processing, artificial vision, detection, driving car, GPS

## Abstract

This paper discusses the overall design and implementation of a video sensor for the detection of risky behaviors of car drivers near previously identified and georeferenced black spots. The main goal is to provide the driver with a visual audio alert that informs of the proximity of an area of high incidence of highway accidents only if their driving behavior could result in a risky situation. It proposes a video sensor for detecting and supervising driver behavior, its main objective being manual distractions, so hand driver supervision is performed. A GPS signal is also considered, the GPS information is compared with a database of global positioning Black Spots to determine the relative proximity of a risky area. The outputs of the video sensor and GPS sensor are combined to evaluate a possible risky behavior. The results are promising in terms of risk analysis in order to be validated for use in the context of the automotive industry as future work.

## 1. Introduction

The problem with road security is a legitimate study area for humanitarian reasons, of public and economic health. Road accidents are one of the main contingencies of the industrial societies and represent an economic and public health aspect that gains major importance in other developing countries.

Traveling is an inherently risky activity, because the movement creates kinetic energy and if an accident or crash happens, the energetic exchange can be harmful to humans and objects involved. Traveling, especially by road, is one of the most dangerous activities people do.

A secure road is the one that recognizes the realities and limitations of the decision making of the human being. This means that the design and administration of the road individually or in combination has to provide a secure ambient to the conductor.

The name “Black-Spot” is given to a place with high concentration of accidents in a point on the road [1]. The road as such, and the option of traveling by car on it, assumes a risky position, nevertheless, whether it is for bad planning or for construction defect, there are geographical spots on the road that meet a large quantity of vial conflicts; a Black-Spot is a geographical spot of a road track, where there has been at least three catastrophic vial events.

For the previous, it is assumed that the proximity and velocity of a Black-Spot, the driver must concentrate his attention on the action of driving the car, avoiding any attitude that provokes a driving distraction. Also, it is recommended to avoid somnolence and tiredness on this point of the road.

The main objective of this work is to equip the cabin of a vehicle with smart technologies that are capable of detecting the driver's behavior (healthy habits against risky behavior in the context of secure driving). To achieve the goal of the project, techniques of artificial intelligence are used as a medium for the detection and characterization of the steering wheel in the cabin of a bus, truck or any other vehicle of transportation alike, in order to delimitate a region of interest (ROI), and then, detect the hands of the driver inside the ROI in order to ensure that both hands are on the steering wheel of the car. Then, offer the driver a visual warning or auditory that allows preventing possible accidents, caused because the driver does not recognize a behavior of insecure conduction.

In order to achieve the efficient detection of the hands and their position in the area of the steering wheel of the car, first the steering wheel's ellipse is detected through 3D reconstruction techniques. An experiment was conducted through the design of a stereo camera using two webcams Microsoft LifeCam model Vx800 for their low cost and ease of use. The last said with the purpose of making the process of detection more efficient since it is critical for the detection and segmentation of the hands afterwards.

Finally, the segmentation of the hands is achieved through skin detection techniques, assuming it is a human being the one driving the car. The image procession is a multiple of the resolution of the camera stereo—320 × 240 pixels—this one situated on the superior part of the driver, 53 cm away from the steer wheel. The method developed allows the freedom of movement for the driver, the detection of the steering wheel and the ellipse formed, and the detection and segmentation of the hands to know the position and quantity of the ROI of the processed frame. After this, the reproduction of a sequence of audio in the speaker alerts the driver about a possible insecure driving behavior in the proximity of a Black Spot.

The audible warning to the driver about his risky driving behavior is preformed through a speaker of 0.5 watts, eight ohms and five centimeters of diameter situated on top of the driver's head. The hardware reproduces audio sequences and converts them to PWM for their output through the speaker in the form of an audible output of voice. The objective of the hardware is to manage the visible and audible alarms. It is intended to allow the alarm to maintain independence with respect to the processing images software, easing the maintenance and diagnosis of errors.

A GPS sensor is connected through a USB serial port to the computer to know the position in real time (one sample per second) of the vehicle and get important processing parameters, such as geo-referenced position, UTC time and speed in Knots by second in order to maintain compatibility between the metric and English measurement system. The data base for Black-Spots has been manually created for now and it has been considered a test drive sampled for test purposes.

Distraction and fatigue being one of the main causes of accidents for drivers, the development of technologies that promote safe driving habits is an area with opportunity for the application of techniques of artificial vision and embedded systems. The bad driving habits caused by distraction lie mainly within the use of the cell phone, management of audio equipment and GPS or adjustment of the cooling and heating equipment. According to the statistics of the National Highway Traffic Safety Administration (NHTSA), in 2009 an average of 448,000 passengers of cars were injured in accidents on the highways of the United States with the cause being the usage of cell phones when driving [2,3]. Because of this, it is important that the driver is keeping both hands on the steering wheel, and if only one hand or none are identified on the steering wheel of the car in an important lapse of time, the driver is warned through a visual and auditory alarm that is incurred upon risky driving behavior.

The study of incidents and statistics was based in Mexico, and specifically in Colima city, due to the fact that it is a part of the agreement of collaboration of the University Rey Juan Carlos (Spain) and the University of Colima (Mexico) on the development of technologies of vial security, an area in which the group of investigation on Face Recognition Artificial Vision (FRAV) have experience.

### 1.1. Antecedent and Facts about Vial Security

Mexico occupies the seventh place worldwide and third regional in death quantity mainly caused by vial accidents, according to the statistics of the Ministry of Health of the Mexican government, with 24,000 deaths a year, of which 4000 are children under 14 years.

The car crashes are caused for many different things; nevertheless distraction and tiredness are the main causes in the actual time, making the use of the cell phone when driving one of the main distraction causes. The Table 1 describes the quantity of accidents that occurred in Mexico from 2004 to 2010.

On the other hand, about the cause of the accidents, the statistics obtained from Mexican Association of Insurance Agencies (Mexico) concludes that a 70% of the vial incidents are due to human mistakes, meaning the driver's fault. Table 2 describes a resume of the data from the year 2007.

However, similar statistics are given in the United States and Europe, according to studies reflected in the official web site for driving distraction from the government of the United States. Being distracted while driving is any activity that can drive the attention of a person out of the chore of driving. All of the distractions, the driver, passenger and the security of third parties [4]. These kinds of distractions include using the cell phone to talk, eating and drinking while driving, talking to the passengers, cleaning the interior, reading maps, watching a video, and adjusting the radio, CD player or MP3. Nonetheless, due to the fact that texting on the cell phone requires visual, mental and manual attention of the driver, this is considered to be the most dangerous.

### 1.2. Related Work

Currently, there are several products on the market whose function is the detection of hands on the steering wheel of a car. There are also articles published in scientific journals and congress. However, none of them deals with the integration of GPS and the study of risks posed by hazardous driving behaviors in the vicinity of black-spots. In [5], IEE offers on its website a solution with fully invasive cap-sense technology, which can be used to detect if the hand is not present on the wheel. Our system will be able, in the next stage, to follow the movement of the hand to know what happens when the hand is not on the wheel. It is possible that the hand is not on the steering wheel because it is performing some other task that is important in driving (changing gear, operate any device, etc.). In the case of [5]the device is glued on the wheel—it cannot tell where the hand is—but a vision-based system could do that.

Ohn-Bar and Trivedi in [6,7], shows the most significant results of 2014. The article shows a different way to do what we do, because what is discussed are hand gestures. They have a set of dataset-gestures and the system interprets what the driver does. However, it is not good for driving “naturist”. We want the driver to drive the car and we later assess the risk.

### 1.3. Structure of the Article

The goal of the present paper is to provide a comprehensive detection method of full ellipses in the steering wheel of a car, since this means that the driver has removed his hands from the steering wheel. Most projects focus on drowsiness or driver distraction as opportunity areas for artificial vision to provide a visible or audible alarm, with the ability to attract the attention of the driver, and promote good driving [8]. It is something that has been underserved by scholars. This article is divided into four sections, the first one of them is introductory, the second one talks about the methodology used to achieve the objectives, the third one talks about the software and the obtained results, finally the conclusions expose briefly comments related to the results and the lessons learned while developing this project.

## 2. Methodology

### 2.1. Introductory Part

**Figure 1.**
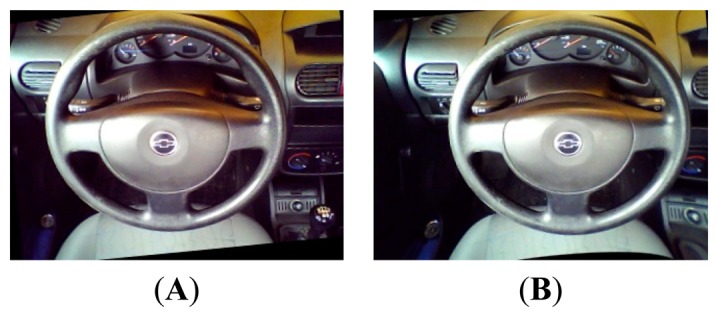
Images from testing samples in cabin. (**A**) Left camera (**B**) Right camera.

For the purpose of testing the hardware platform, several image samples were taken in the cabin in various lighting scenarios. Samples can be seen in Figure 1; the last one is the final version rectified and remapped. For every sample, two images were taken, right and left, as the camera is stereo vision.

**Figure 2.**
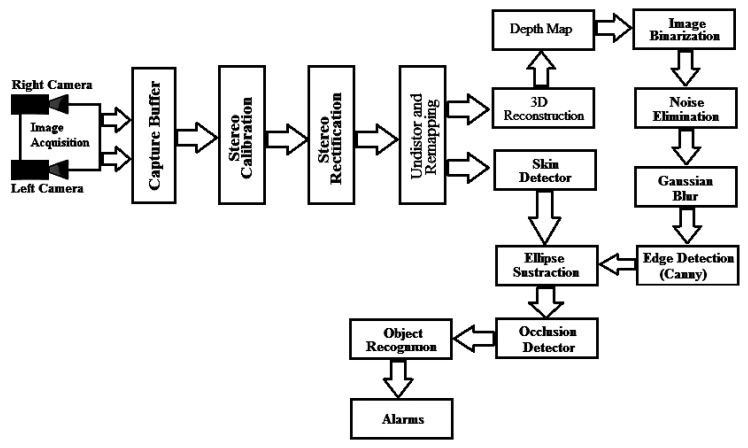
Schematic of vision system.

Figure 2 shows the schematic of the implementation. The firmware solution was developed in C++ using the OpenCV suite version 2.3.2 for the vision part.

### 2.2. Stereo Camera

**Figure 3.**
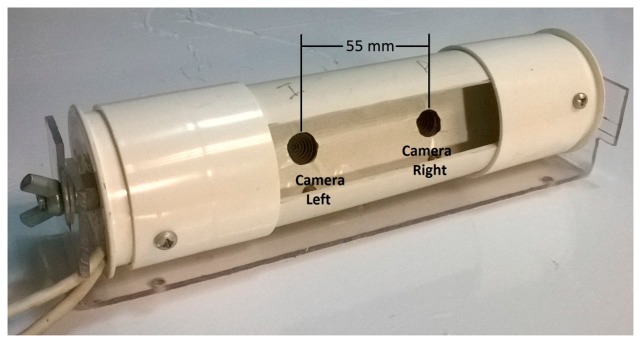
Stereo camera.

The main part of the video sensor system is the stereo camera. In Figure 3 can be seen in detail the camera that was built. For the implementation, two webcams Microsoft LifeCam, model Vx800, were used with a resolution of 640 × 480 pixels and a CMOS sensor of 0.31 MP with autofocus.

A stereo vision system is designed to extract 3D information from digital images and use these for examining the position of objects in two images, to build an advanced object recognition system that recognizes objects in different arrangements (for example, when objects are placed one in front of the other), tracking different objects, *etc*.

Because a stereo vision is similar to the human biological system, some of the features are identical. For example, a human has two eyes to see slightly different views of the same environment. A stereo vision system has two cameras located at a known distance which take pictures of the scene at the same time. Using the geometry of the cameras, we can apply algorithms and create the geometry of the environment.

The model of stereoscopic vision is important for the obtainment of the form of an object in a scene. It constitutes an important technique for the calculus of the distance of the pixels inside of a frame of an image. In [9,10]it is discussed extensively the model of the camera pin hole, as well as the complete methodology.

The stereo vision is one of the main topics in the investigation of the computer vision; an image can be described better having two points of view, each one obtained from two cameras situated at a certain distance from each other. The comparison of the distances from the object to the camera allows the calculus of the so-called depth map. A depth map is a 2D image in which the color of every pixel represents the distance from that point to the camera. In other words, the pixels of light are close and the dark pixels are far.

The above diagram contains equivalent triangles. Writing their equivalent equations will yield us the following result: [10]

(1) $$\text{disparity}=X-X\prime =\frac{Bf}{Z}$$

**Figure 4.**
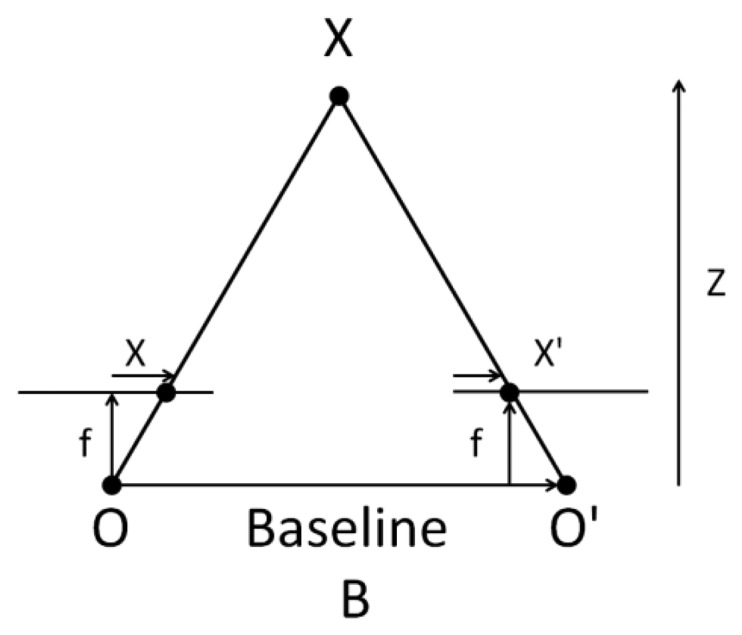
Pin hole camera model.

The distance between X and X′ points, shown in Figure 4, is the image plane corresponding to the scene point 3D and their camera center. B is the distance between two cameras (which we know) and f is the focal length of camera (already known). So in short, the above equation says that the depth of a point in a scene is inversely proportional to the difference in distance of corresponding image points and their camera centers. So with this information, we can derive the depth of all pixels in an image.

In the modeling of an image for the obtaining depth map, the following techniques are involved:

- Camera calibration
- Rectification of left and right images
- Remapping and undistorted
- Depth map calculation

**Figure 5.**
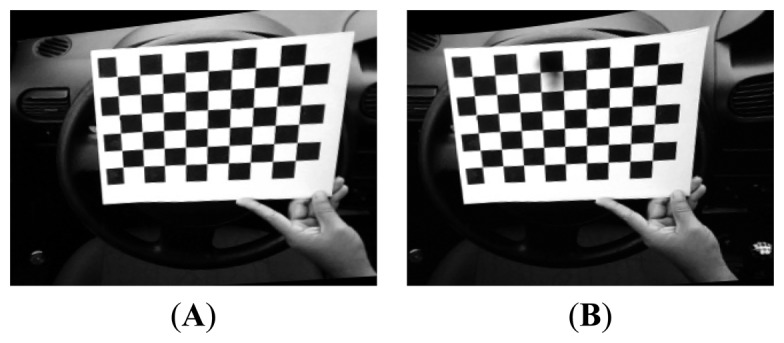

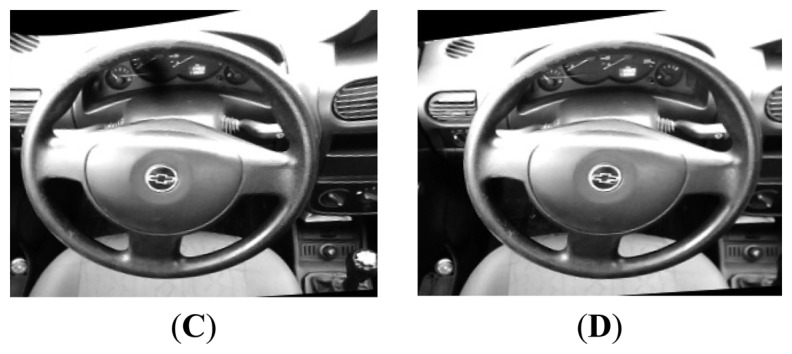
Camera calibration. (**A**) ChessboardL (**B**) ChessboardR (**C**) Rectified and remapped left (**D**) Rectified and remapped right.

The calibration process is one of the most important parts in the processing of stereoscopic images; the attainment of the intrinsic and extrinsic camera parameters is required for further processing in real time video frames, and this calculus is to produce 3D images or movies, or to carry out the calculation of depth maps in order to identify plans within a scene. Figure 5 describes the process carried out according to Wang [11].

The algorithm Block Matching (BM) to obtain a disparity map is one of the fastest and simple to implement oriented regions.

### 2.3. Ellipse Detect and Segmentation

The detection of ellipses is not as studied in the field of artificial vision, but is a major field of study in the medical area. A detailed study of the implementation of the Hough Transform is described by Lu and Tan in [12]. However, the nearest source of this project is the Cabintec project [13], in which the detection of ellipses is part of the subproject Alert CABINTEC. However, the issue of detection of risky driving behaviors by drivers is not a new area of research. In [14,15]there are similar projects described.

The Hough Transform is mostly used as a basis for the detection of ellipses and circles in stills taken from video frames [16–28], being the technique specifically proposed by Fitzgibbon, *et al.* [21], and studied in this paper to detect the ellipse formed by the wheel of the vehicle.

OpenCV has a nice in-built ellipse-fitting algorithm called fitEllipse, it uses least-squares to determine the likely ellipses [29]. Some tasks are involved in the detection of an ellipse or a family of ellipses within a scene, such as:

- Enhancement and image filtering
- Smooth (Gaussian Blur)
- Binary threshold or InRange
- Border Filter Detect (Canny or Sobel as needs)
- Contour segmentation
- Ellipse detection

### 2.4. Skin Segmentation

A very effective method in the segmentation of image elements related to human beings is the detection of tonalities or similar textures to human skin. The detection of skin is one of the mostly used techniques to segment portions of scenes where there are usually a lot of probabilities of finding skin colored tonalities, such as faces or hands.

Elgamal and Muang in [30] establish that skin detection is the process of finding skin-colored pixels and regions in an image or a video. This process is typically used as a preprocessing step to find regions that potentially have human faces and limbs in images. Several computer vision approaches have been developed for skin detection. A skin detector typically transforms a given pixel into an appropriate color space and then uses a skin classifier to label the pixel whether it is a skin or a non-skin pixel. According to this, as a result of segmentation, we will obtain a scene with pixels that are candidates with a strong ownership of skin tonality, and this way, apply the algorithms of face or hands recognitions, respectively.

There has been some disagreement between the authors in respect to which is the proper space color for the RGB conversion of the original frames. In some cases, the classification in HSV space color seems to be the right one; however, it led to resulting images with tending red colored tonalities. In other cases, according to the classification of the races, it seems like the space of color YCrCb allows segmented images with a better pixel speed and a strong tendency to a skin like color which is more compatible with the detector.

**Figure 6.**
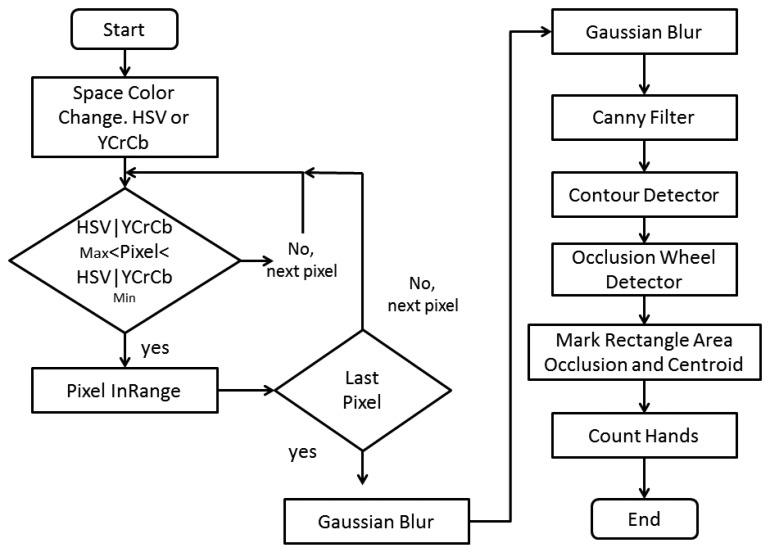
Skin detection algorithm.

In the present project, it is required to segment through a skin detector those pixels that correspond to a skin color, to later detect occlusions belonging to the hands on the steering wheel. Figure 6 describes the algorithm.

### 2.5. Algorithm of Artificial Vision

**Figure 7.**
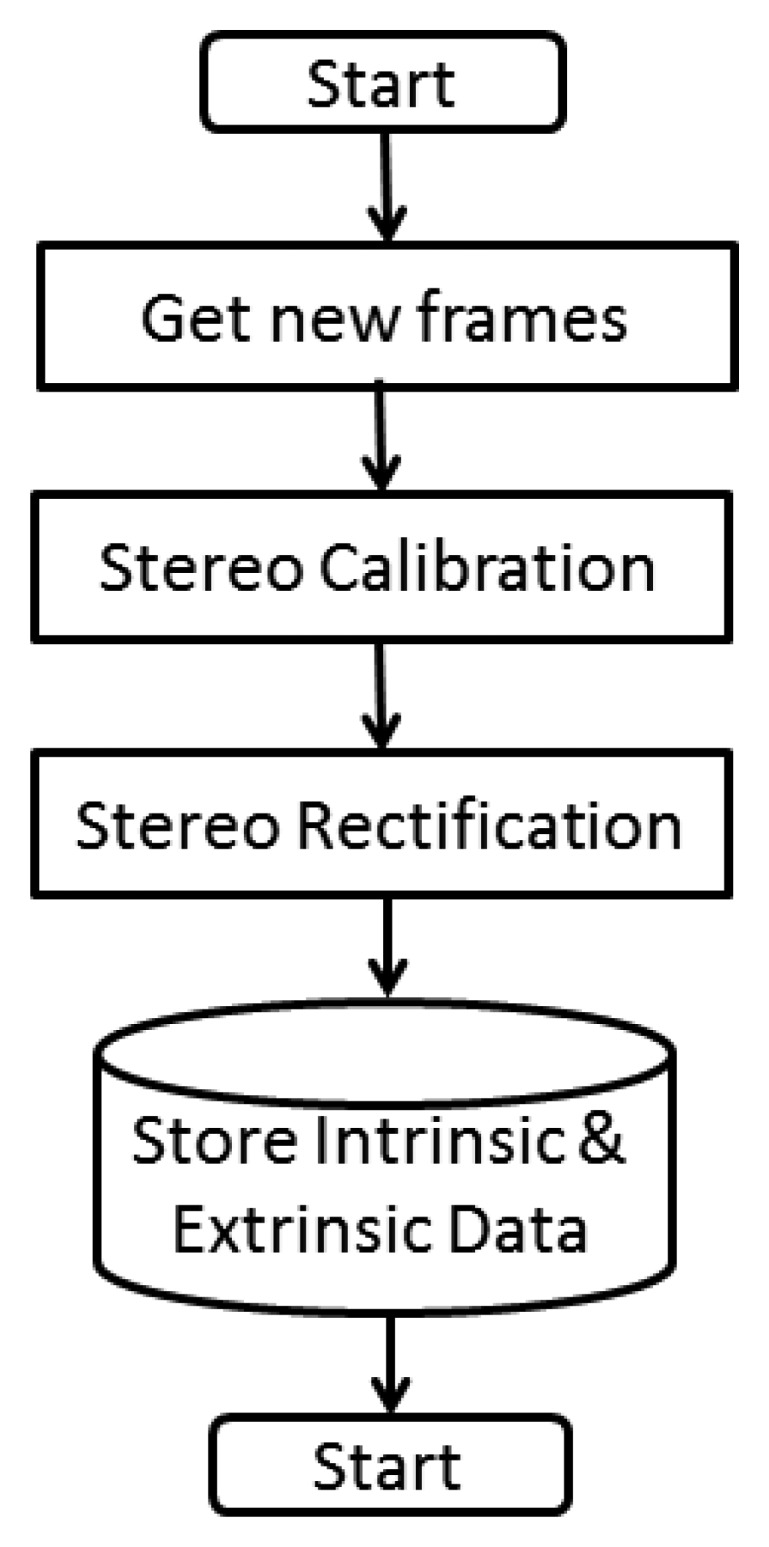
Flow chart initial stage.

In developing the firmware that implements the algorithm, we will try to maintain as much simplicity and accuracy as possible. Figure 7 describes in detail the algorithm codified in the firmware for the initial stage, the calibration and extraction of intrinsic and extrinsic data.

**Figure 8.**
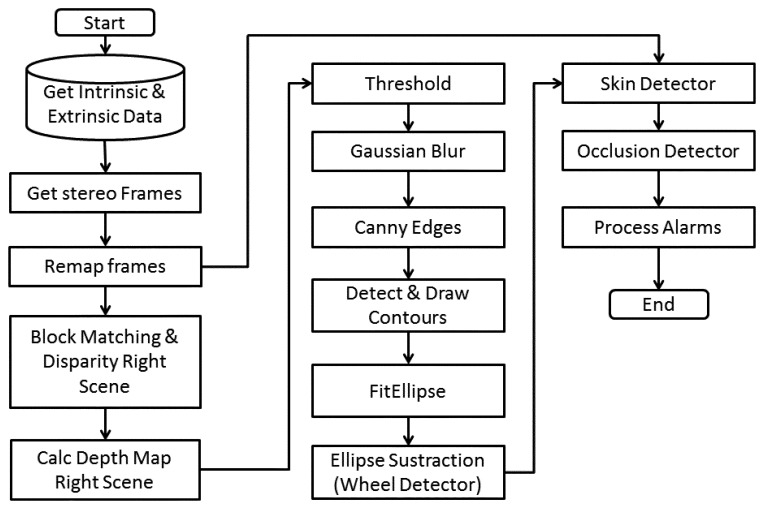
Flow chart alarm mode operation.

When the system is operating in real time, the Figure 8 describes the normal operation in alarm mode. Due to the timing requirements, there is just a 16.66 ms image processing window. Due to this, the display is used just for parameters and driver messages, and not for image debugging.

### 2.6. Hardware Platform

**Figure 9.**
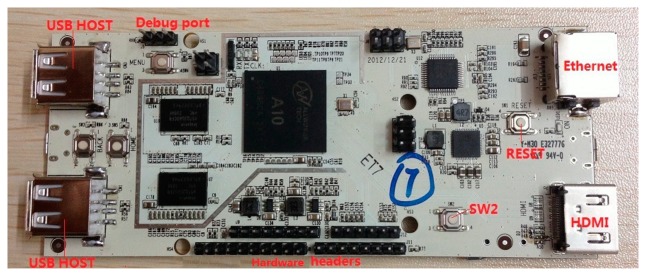
pcDuino mini PC platform.

The heart of the design is the microcontroller. The ARM architecture of the microcontroller allows a more robust and better performing firmware development. The two development platforms are Ubuntu Linux and OpenCV for ARM port. We decided to implement the firmware on C++ due to the natural integration on a PC platform and the richness of free software routines in the community of artificial vision internet. The design of the base platform is like that in Figure 3. Another reason for using C++ is the natural integration with Eclipse IDE, which allows natural migration to a windows system. Later, with solution generation and debugging, we can deploy the A10SoC microcontroller via a remote terminal session. The Figure 9 shows the board in detail and Table 3 its hardware specification.

**Figure 10.**
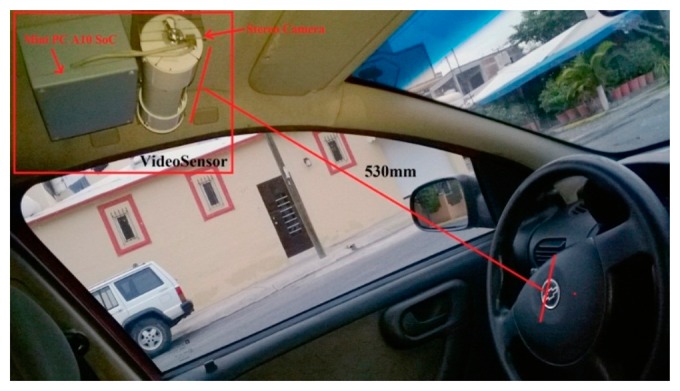
Video sensor platform installed.

Finally, we built a video sensor with the embedded computer platform shown in Figure 9 with the stereo camera integrated and installed in a cabin of a mini truck Chevrolet Tornado, the Figure 10 shows the video sensor in its place.

### 2.7. GPS Platform

The heart of the detection system is the GPS sensor GS405 by SPK Electronics Co. The SPK-GPS-GS405 is a Global Position System receiver module based in SiRF Star III high sensitivity chipset solution which includes a built-in Sarantel omni-directional Geo-Helix SMP passive antenna. The receiver module can track 20 satellites simultaneously; the integration with GPS receiver and the microcontroller computer is carried through the use of a connection RS232C and a transceptor transistor-transistor-logic to USB.

**Figure 11.**
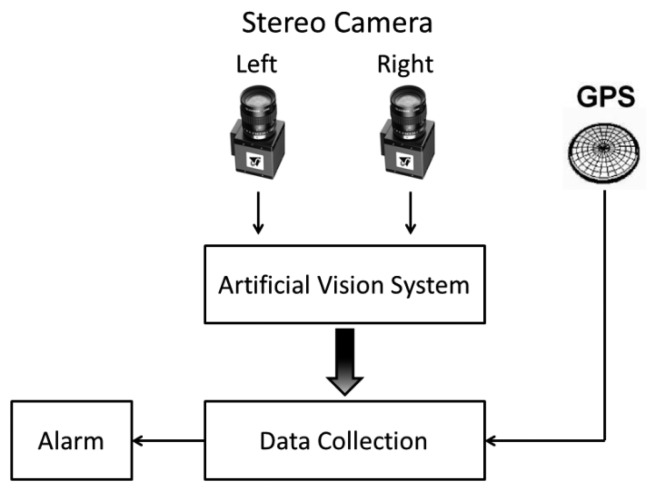
GPS relationship between artificial vision and GPS module.

The data frames are obtained via USB in protocol NMEA0183; they are later filtrated to decode only the message GPRMS or Recommended Minimum Specific GNSS data. Table 4 describes the format of the message RMC, and Figure 11 describes the relation between the vision system and the GPS module.

### 2.8. Risk Analysis

To perform the experiment of risk classification in driving, different signals of risk reference when driving have been generated through the categorization of the risk evaluations in a series of tags that will allow feeding the classifiers considered to generate an alarm. For that, on a first approach, three tags were defined, dividing the rank of the evaluations in three groups: High risk, Medium risk and Low risk.

In this case, *y_i_*(*t*) being the established risk by the expert *i* on the instant *t* the risk tag *R_i_* for the instant *t* is given by:

(2) $$R_{i}\left(t\right)=\left\{\begin{array}{rr} \text{Low Risk} & if0\leq y_{i}\left(t\right)\leq 33,\\ \text{Medium Risk} & if33\leq y_{i}\left(t\right)\leq 67,\\ \text{High Risk} & if67\leq y_{i}\left(t\right)\leq 100, \end{array}\right\}$$

At the present time, the evaluation of the risk situations in real sceneries is still on course. The Table 5 shows the main risk situations to evaluate according to the action being done by the driver.

## 3. Software and Results

### 3.1. Image Processing

For the testing, the imaging camera is done by taking 5.36 frames per second, the overall performance of the processing stage of the image is 177.62 ms, since the human eye has a real time resolution of 22–24 frames per second (image). It can therefore be said that the system is in real time. The entire test was performed in a graphics terminal for testing purpose.

**Figure 12.**
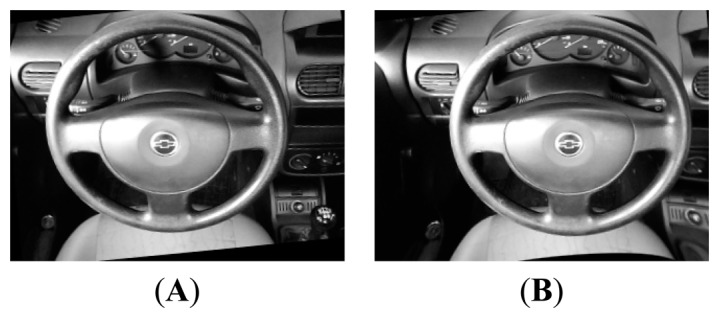

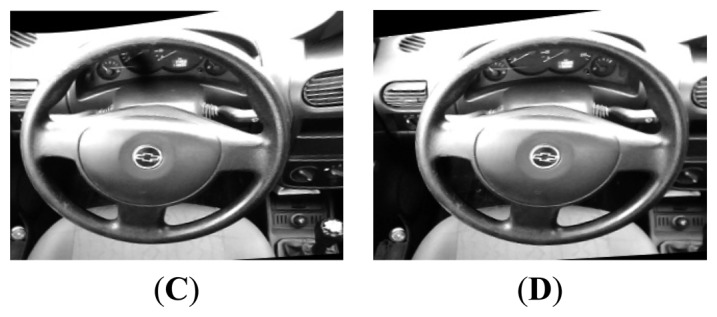
Image in stereo (**A**); Left not remapped (**B**) Right not remapped (**C**) Left remapped (**D**) Right remapped.

In this section, the filter is applied to gray, and is very important for imaging because it is responsible for converting the images obtained to 8-bit tones, this being necessary for the function object detection. The Figure 12 shows the parameters from Table 7 and main results of the system of vision.

Below in Table 6, a list of lighting levels for which video samples were made is shown, as well as the phase of day when the sample was taken and the experimental results.

**Figure 13.**
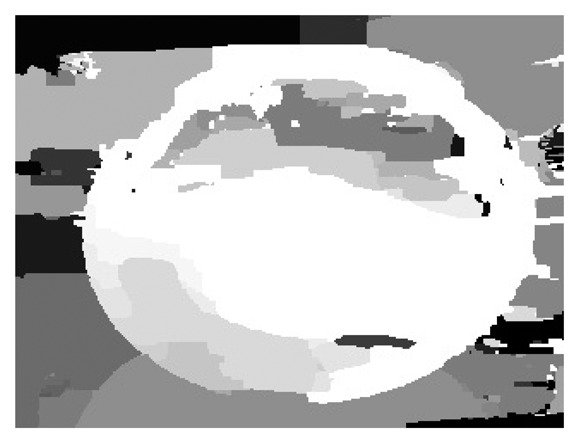
First stage depth map.

After the process of remapping the images, it is possible to calculate the depth map for the detection of the ellipse that conforms to the steering wheel from the cabin of the car. Figure 13 shows the resultant image.

### 3.2. Canny Edge Detection

**Figure 14.**
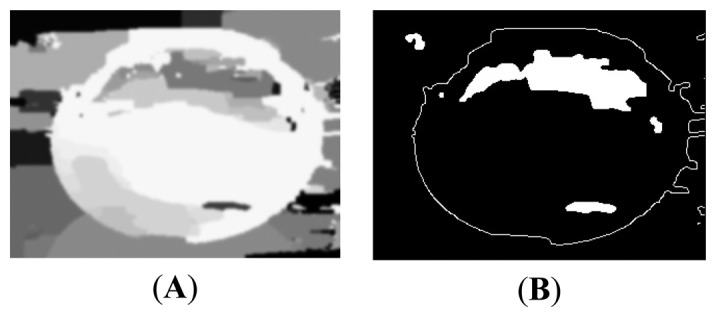
Filters (**A**) Smooth image (**B**) Threshold image.

The application of noise filters is essential before subjecting the image segmentation in Canny filters, to obtain more clarity, and so that the object recognition process with the segmentation algorithm based on borders (Hough Transform) is more precise. They are applied to two noise filters: threshold and smooth. In Figure 14A,B can be seen the images resulting from the application of filters.

### 3.3. Ellipse/Circle Detection

**Figure 15.**
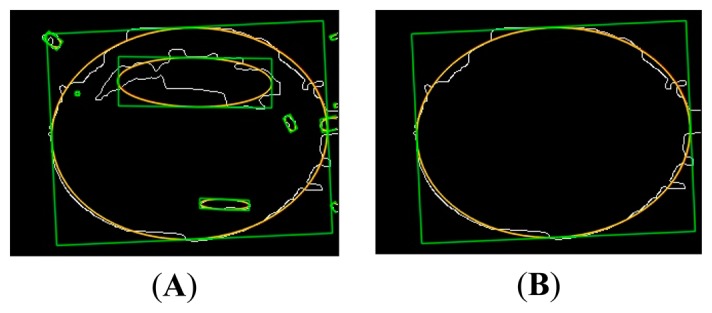
Ellipse/circle detection (**A**) Family ellipses detected (**B**) Steering wheel.

Subsequently, the detection of the objects takes place by the application of noise filters and the object detection filter algorithm (Hough Transform). This is responsible for detecting the object—the automotive steering wheel—having as output a contour indicating the detected object. Hough-ellipse detects a family of ellipses, this due to the efficiency of the algorithm, the biggest one is the one that belongs to the steering wheel of the car's cabin. In Figure 15A,B the last steps can be seen.

**Figure 16.**
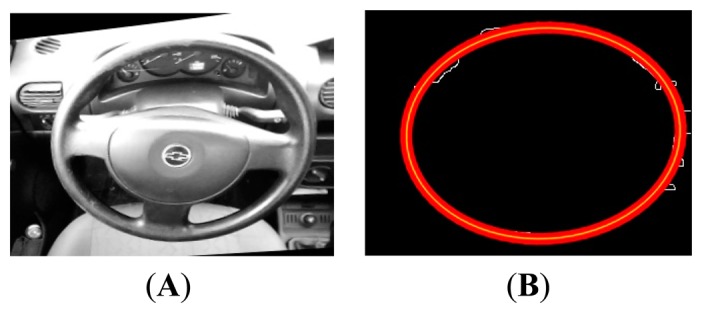

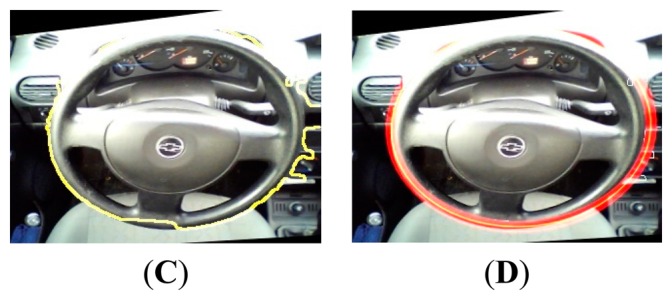
Ellipse segmentation final (**A**) Original (**B**) Contour (**C**) Ellipse (**D**) Image add.

Finally, the sequence of Figure 16A–D shows the final result of the segmentation of the ellipse formed by the wheel of the car. Also, the result of the sum to the original image detected is shown.

### 3.4. Hands Segmentation and Enumeration

**Figure 17.**
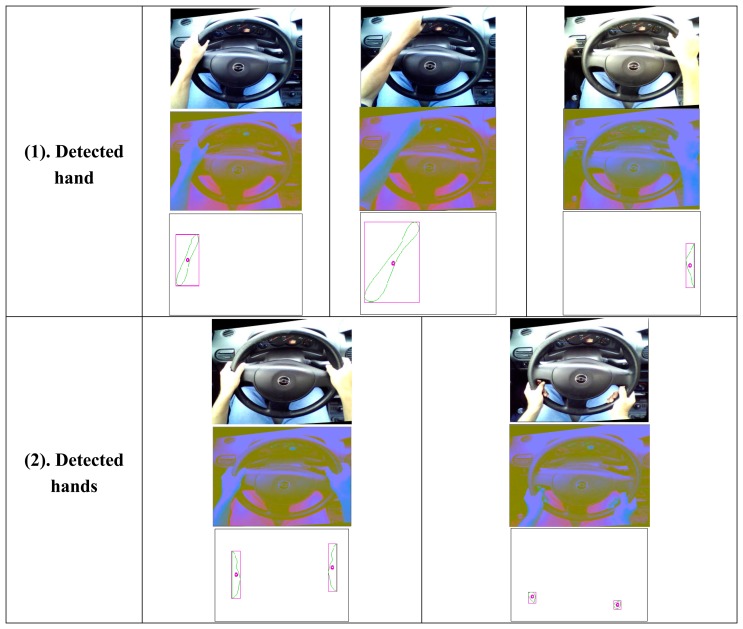
Hands enumeration.

The results of the application of the final part of the algorithm are the segmentation for skin detection and the detection of the number of occlusions that intersect with the ellipse detected in the steering wheel. The experiment was carried out with 2000 frames of video effectuated on the frame obtained from the camera on the right, since it is not necessary to conduct tests on both frames. The sequences in Figure 17 shows the families of images that were found; for original image RGB, YCrCb image and enumeration with calculated centroid.

### 3.5. Limitations and Scope of the System

The results presented are valid for the range of illumination of 1769–1024 lux, in the hours between 2 and 4 pm, see Table 2; measured at the center of the Pacific Time-Zone (UTC -6). This range of lighting was determined as the optimum. At the time of evaluation of the manuscript, we conducted overnight tests with two IR illuminators (one for each camera), which were Marubeni brand and model L850-66-60-550, at 7.5 watts, with 850 nM for lighting CCD. Because the position of the hands on the wheel is determined by the ellipse formed by the steering wheel, the system can automatically find their position, and center coordinates, and the above changes in your position require this to be recalibrated. The calibration of ellipse steering is automatic, and then the system can be adapted to any type of car.

## 4. Conclusions

In this paper, the results of evaluation of a set of algorithms for the detection of the steering wheel inside the cabin of a car and the enumeration of the hands using the steering wheel are given. The latter is to alert the driver of the car at risk of committing risky driving behavior.

The artificial vision is a discipline of artificial intelligence with various industrial applications. At present, given the advent of the use of microcontrollers, it has emerged as an area of opportunity for embedded machine vision.

While the development of machine vision application resources can be considered unlimited, in a PC environment under the microcontroller, the design is not.

In the particular application, a hardware platform running at 1 GHz should be considered for development work. One of the reasons is to work with images at a resolution of 320 × 240 pixels and to achieve a speed of processing like real time.

In future work, we would recommend eliminating the integrated graphical environment for a final solution to the maximum of 200 frames per second so that the camera sensor can be used at full capacity.

## Figures and Tables

**Table 1.** Quantity of vial incidents on highway in Mexico. According to the statistics of the National Geographic Institute of Statistics and Informatics (INEGI, Mexico).

**Cause of the Accident**	**Year of Occurrence**						

	**2004**	**2005**	**2006**	**2007**	**2008**	**2009**	**2010**
Driver	303,516	339,409	343,248	377,724	381,293	384,942	389,026
Pedestrian or passenger	4,041	4,485	4,310	4,011	3,975	4,773	4,573
Vehicle malfunction	5,196	5,720	6,161	2,998	3,519	4,337	4,181
Bad road condition	32,706	4,232	3,376	3,370	3,785	4,446	4,748
Other	98,148	98,387	114,177	88,176	73,863	29,969	24,739

**Total**	**443,607**	**452,233**	**471,272**	**476,279**	**466,435**	**428,467**	**427,267**

**Table 2.** Main causes of vial incidents in Mexico in the year 2007. According to Mexican Association of Insurance Agencies.

**Causes of Vial Incidents on Cars**	

**Global percentage (%)**	**Description**
7	Car malfunction
8	Truck malfunction
15	For bad weather
70	Caused by the driver

**70% Caused by the Driver**	

**Percentage (% on 70%)**	**Description**

7	Fatigue
7	Bad overtake
8	Bad common sense
8	No respecting vial signals
20	Doing drugs or drinking alcohol when driving
20	Vehicular scope
30	Overspeed

**Table 3.** AllWinner A10SoC mini PC platform. [30]

**Hardware Specification**	

**Item**	**Detail**

CPU	AllWinner A10 SoC, 1 GHz ARM Cortex A8
GPU	OpenGL ES2.0, OpenVG 1.1 Mali 400 core
DRAM	1 GB DDR3
OnBoard Storage	2 GB Flash, macro SD card slot for up to 32 GB
Video Output	HDMI 720 p or 1080 p 60 Hz
OS	Linux3.0 + Ubuntu12.10
Extension Interface	Arduino Headers
Network Interface	Ethernet 10/100 MBps RJ45
Power	5 V/2 Amp
USB	2 Host, 1 OTG

**Table 4.** RMC—recommended minimum specific GNSS data.

$GPRMC, 161229.487, A, 3723.2475, N, 12158.3416, W, 0.13, 309.62, 120598, E, *10			

**Name**	**Example**	**Units**	**Description**
Message ID	$GPRMC		RMC protocol header
UTC Time	161229.487		hhmmss.sss
Status	A		A = data valid or V = data not valid
Latitude	3723.2475		ddmm.mmmm
N/S Indicator	N		N = north or S = south
Longitude	12158.3416		dddmm.mmmm
E/W Indicator	W		E = east or W = west
Speed Over Ground	0.13	knots	
Course Over Ground	309.62	degrees	True
Date	120598		ddmmyy
Magnetic Variation ^1^		degrees	E = east or W = west
Checksum *10			
<CR><LF>			End of message termination

1 All “course over ground” data are geodetic WGS84 directions.

**Table 5.** Major risk situations detected in conducting.

**Description of the Detected Situation**	**Persistence (s)**	**Case of Usage**	**Weight**
Mobile phone usage (answering calls)	6	1 hand	0–33
Mobile phone usage (making a call)	3	1 hand	67–100
Mobile phone usage when changing lane	5	1 hand	67–100
Hand gestures when speaking to the passenger	5	0 hands	67–100
Mobile phone usage when changing gears	6	1 hand	33–67
Mobile phone usage when turning	3	1 hand	67–100

**Table 6.** Lighting levels for video and number of samples taken.

**Level (luxes) Car-Indoor**	**Time of Day (UTC-6)**	**#Samples**	**Detected**
1769	Midday (2 PM)	1500	1486
1024	Sunset (4:30 PM)	500	454
638	Early morning (10 AM)	100 for testing	0
334	Night fall (6 PM)	100 for testing	0
78	Early night (7:30 PM)	100 for testing	0

**Table 7.** System parameters.

**Final Parameters**				

**Component**	**Detail**			
FPS total	5.36			
Calibration stereo time	7.65 s			
Disparities in BM algorithm	48, due the webcam is autofocus			
#iters in BM algorithm	4			
Max Ellipse Detected	7			
Image Database for Wheel Ellipse Detection, Depth Map	385, Right Camera			
Image Database for Hands Detection	2000, both Left and Right Camera			

**Results of the Experiments in Artificial Vision**				

**Depth Map Max. Time Processing**	**458 ms**			

	**Accuracy**	**#Samples**	**Detected**	**False Negative**

% Image Ellipse Detected	96%	200	192	8
% Hands Positive Detected	97%	2000	1940	60
